# Ancient peat and apple extracts supplementation may improve strength and power adaptations in resistance trained men

**DOI:** 10.1186/s12906-016-1222-x

**Published:** 2016-07-18

**Authors:** Jordan M. Joy, Roxanne M. Vogel, Jordan R. Moon, Paul H. Falcone, Matt M. Mosman, Zbigniew Pietrzkowski, Tania Reyes, Michael P. Kim

**Affiliations:** Department of Nutrition and Food Sciences, Texas Woman’s University, Old Main Building 307, PO Box 425888, Denton, TX 76204-5888 USA; School of Health Sciences, American Public University System, Charles Town, WV USA; MusclePharm Sports Science Institute, MusclePharm Corp., Denver, CO USA; Maximum Mobile Fitness, Spearfish, SD USA; FutureCeuticals Inc., Irvine, CA USA; Anschutz Medical Campus, University of Colorado, Denver, CO USA

**Keywords:** Ergogenic aid, Mitochondria, Performance, ATP, Sport nutrition

## Abstract

**Background:**

Increased cellular ATP levels have the potential to enhance athletic performance. A proprietary blend of ancient peat and apple extracts has been supposed to increase ATP production. Therefore, the purpose of this investigation was to determine the effects of this supplement on athletic performance when used during 12 weeks of supervised, periodized resistance training.

**Methods:**

Twenty-five healthy, resistance-trained, male subjects completed this study. Subjects supplemented once daily with either 1 serving (150 mg) of a proprietary blend of ancient peat and apple extract (TRT) or an equal-volume, visually-identical placebo (PLA) daily. Supervised resistance training consisted of 8 weeks of daily undulating periodized training followed by a 2 week overreach and a 2 week taper phase. Strength was determined using 1-repetition-maximum (1RM) testing in the barbell back squat, bench press (BP), and deadlift exercises. Peak power and peak velocity were determined during BP at 30 % 1RM and vertical jump tests as well as a 30s Wingate test, which also provided relative power (watt:mass)

**Results:**

A group x time interaction was present for squat 1RM, deadlift 1RM, and vertical jump peak power and peak velocity. Squat and deadlift 1RM increased in TRT versus PLA from pre to post. Vertical jump peak velocity increased in TRT versus PLA from pre to week 10 as did vertical jump peak power, which also increased from pre to post. Wingate peak power and watt:mass tended to favor TRT.

**Conclusions:**

Supplementing with ancient peat and apple extract while participating in periodized resistance training may enhance performance adaptations.

**Trial Registration:**

ClinicalTrials.gov registration ID: NCT02819219, retrospectively registered on 6/29/2016

## Background

Adenosine-5’-triphosphate (ATP) and ATP metabolites are involved in numerous biological processes including cardiac function, neurotransmission, blood flow, and muscle contraction [1, 2], and it has been suggested that increased ATP levels correlate with improved health and performance [3–5]. Direct supplementation with exogenous ATP has demonstrated divergent results in terms of increasing ATP when measured in whole blood [3, 6, 7]. Therefore, supplementation strategies for increasing endogenous ATP levels may be desirable. Previously, oral supplementation with a proprietary blend of ancient peat and apple extracts have been demonstrated to increase intracellular ATP levels in whole blood and intramuscular levels of ATP in resting subjects, suggesting increased activity of bodily processes that lead to endogenous ATP production [8, 9].

Previous research has found oral supplementation with a proprietary blend of ancient peat and apple extracts to increase ATP concentrations in whole blood of resting subjects as well as intramuscular concentrations in one volunteer [9]. Preliminary reports from this laboratory suggest this occurs without an increase in reactive oxygen species, which may be associated with increased ATP production [10]. In fact, ancient peat and apple extracts may actually decrease reactive oxygen species [8], possibly blunting a potential increase caused by resistance training [11].

Another nutritional supplement that has been well documented as an ergogenic aid via modulation of the phosphagen energy system is creatine monohydrate. While it would be bold to suspect another supplement to match the myriad of athletic performance benefits of creatine at present, it stands to reason that other ATP-enhancing supplements would be the premier candidates. In brief, creatine has demonstrated efficacy for improving maximal strength, peak power, and fatigue resistance [12, 13]. The primary mechanism for creatine’s ergogenic effects is via rephosphorylation of adenosine diphosphate to ATP via creatine phosphate [14, 15]. Thus, the potential exists for ATP increased through alternative means to also increase strength, power, and exercise tolerance.

Despite these observations, supplementation for indirect ATP enhancement is yet to be evaluated for potential to augment performance in response to resistance training. However, the existing data on ancient peat and apple extracts for increasing both whole blood and muscle ATP levels [8, 9] and muscle mass [16] support the plausibility for chronic supplementation yielding positive augmentation of performance following resistance training. Therefore, the purpose of this study is to determine the effects of a proprietary blend of ancient peat and apple extracts on athletic performance. It was hypothesized that supplementation would improve strength and power over the duration of the training program as well as blunt a decrement in performance due to overreaching.

## Methods

### Participants

Twenty-five healthy, resistance-trained, male subjects (28 ± 5y; 176.0 ± 6.5 cm; 83.2 ± 12.1 kg) completed this double-blind study. 33 subjects were recruited, and 3 subjects did not complete the study due to scheduling conflicts, 3 were not compliant with protocols, and 2 sustained injuries during the study unrelated to training or supplementation. All subjects were prohibited from using any supplements not provided in the study except for a multivitamin or protein powder food substitute, which they were not permitted to use within 2 h before or after resistance training sessions. Each subject was required to be capable of lifting 1.5x their bodyweight in the squat and deadlift and 1x bodyweight in the bench press. At baseline, the placebo (PLA) group was able to squat 1.71 ± 0.21, bench press 1.45 ± 0.19, and deadlift 2.17 ± 0.25 times their bodyweight, and the treatment (TRT) group was able to squat 1.66 ± 0.24, bench press 1.31 ± 0.20, and deadlift 1.93 ± 0.27 times their bodyweight. Approval for research with human subjects was obtained from the MusclePharm Sports Science Institute IRB (accredited by the United States Department of Health and Human Services), and protocols conformed to the standards set by the latest revision of the Declaration of Helsinki. No members of the IRB were involved in study conception, design, data collection, data analysis, or data interpretation. Subjects provided their written informed consent prior to participation in the study.

### Experimental design

Subjects were randomly assigned to either the PLA (*n* = 11) or TRT (*n* = 14) groups. They were instructed to consume 1 serving (2 mL) of either PLA or TRT (elevATP®, VDF FutureCeuticals Inc., Momence, IL; 150 mg) 45 min prior to training on training days or at a similar time of day on rest days. The supplement was provided as a liquid with liposomes (QuSomes, BioZone Laboratories Inc., Pittsburg, CA), and the instructed dose was marked on the dropper provided with the vial. PLA consisted of a flavor-matched liquid with identical liposomes added. A third-party, BioZone Laboratories Inc., assembled the TRT and PLA supplements and provided disclosure as to their exact composition to verify subjects received the supplement as described and nothing more. Despite this assumption, the ingredients were not independently verified, and thus, exact contents cannot be confirmed. Supplement vials were weighed to ensure compliance. Subjects were resistance trained under the guidance of a certified strength and conditioning specialist 3 days per week for 8 weeks followed by a 2 week overreach and 2 week taper phase corresponding to weeks 9–10 and 11–12, respectively, in a design identical to that previously described [16]. A eucaloric diet consisting of 50 % calories from carbohydrates, 25 % from protein, and 25 % from fat was prescribed to all subjects at the onset of the study, and diets were tracked weekly via 3-day food logs. Total calories were determined for each individual based on the Mifflin St. Jeor equation adjusted for activity level [17]. Subjects were measured at weeks 0, 4, 8, 10, and 12 for all performance variables. Variables collected consisted of upper and lower body power, upper and lower body maximal strength, maximal and average anaerobic power, upper and lower body strength endurance, irisin, interleukin-6 (IL-6), IL-15, fibroblast growth factor-21 (FGF-21), myonectin, cortisol, C-reactive protein (CRP), and growth differentiation factor-11 (GDF-11). Blood draws were conducted at weeks 0, 4, 8, and 12.

### Resistance training program

The resistance training program performed by all subjects has been previously reported [16]. Briefly, weeks 1–8 (standard resistance training phase) consisted of one muscle hypertrophy-oriented workout, one power- oriented workout, and one strength-oriented workout featuring cycle ergometer Wingates following strength training. The back squat, bench press, and deadlift exercises were performed on each day along with several other multi- and single-joint exercises. Participants rested 48–72 h between each training day. During the overreach phase (weeks 9 and 10), participants performed high volume workouts on Monday through Thursday with a strength-oriented workout or performance testing conducted on Friday for weeks 9 and 10, respectively. The taper phase (weeks 11 and 12) consisted of one power day on Mondays then strength and power days on both Wednesdays and Fridays performed at low volume for back squat, bench press, and deadlift only. One serving (35 g) of a whey protein supplement (Combat, MusclePharm Corporation, Denver, CO) providing 25 g of protein, 5 g of carbohydrate, and 1.5 g of fat was provided to all subjects immediately following exercise on all training days.

### Measurements

Measurements rotated between upper and lower body exercises to provide localized rest (about 15–20 min), and they were conducted in the order which they are presented herein. Maximal strength was determined using 1-repetition maximum (1RM) tests in the barbell back squat, bench press (BP), and deadlift exercises. Subjects were required to descend such that the anterior hip crease descended below the top of the knee during the squat 1RM test, to make contact with their chest without bouncing or removing their hips from the bench during the BP 1RM test, and they were prohibited from hitching motions in the deadlift. Total strength was calculated as the sum of squat, BP and deadlift 1RMs. All 1RMs were monitored by a Certified Strength and Conditioning Specialist who is also a competitive powerlifter. Lower and upper body power was determined using a linear force transducer (weightlifting analyzer, TENDO Sport Machines, Slovak Republic) during a vertical jump and BP test. The greatest value out of 3 tests for jump height, peak power, and peak velocity were recorded while using a Vertec to measure height. Upper body power was determined via the BP exercise at 30 % 1RM. Subjects performed 3 sets of 3 repetitions under the same rules as the 1RM test. The greatest value for peak power and peak velocity was recorded. Strength endurance was determined using 50 % of each subject’s 1RM and by having them perform repetitions until reaching muscular failure in the squat and bench press exercises. A repetition was subtracted if the participant rested greater than 1 s at the top for squat or at the top or bottom for bench press. Repetitions performed were recorded, and repetitions x load was used to calculate total work performed. Anaerobic power output was determined using a 30s Wingate anaerobic cycle ergometry test (WattBike, Woodway, Waukesha, WI). Each Wingate test consisted of 1 min of light pedaling (50–60 rpm), a 5 s sprint, 2 min of light pedaling, another 5 s sprint, and another 2 min of light pedaling all against no resistance prior to the 30s test. Seat height was recorded during the first visit for each participant and kept constant for every measure. Subjects were provided strong verbal encouragement throughout the test. Peak power, average power, watt:mass, and average speed were recorded. Measurements were conducted at baseline and repeated following weeks 4, 8, 10, and 12. The week 8, 10, and 12 measurements were taken corresponding to the end of the standard resistance training, over reach, and taper phases, respectively. Test-retest separated by 7 days resulted in an intraclass correlation coefficient > 0.965 for all measures.

### Serum analysis

Blood draws were performed via venipuncture by a trained phlebotomist. Following a 10-h fast, all subjects submitted a blood sample for analysis in the morning to control for diurnal variations. Blood was drawn from the antecubital vein into dry serum tubes (Vacutainer, Becton, Dickinson and Company, Franklin Lakes, NJ). Upon clotting, blood was centrifuged and serum was collected for analysis. Serum was centrifuged at 5000 g for 5 min to pellet debris. Irisin (Biovision, Milpitas, CA); myonectin (Aviscera Bioscience Santa Clara, CA); GDF-11 (San Diego, CA); cortisol (Spring Valley, CA); FGF-21, IL-6, IL-15, and CRP (R & D Systems, Minneapolis, MN) were measured using quantitative sandwich ELISA kits, following the instructions provided for each kit. Final reactions were measured using a spectrophotometer (Molecular Devices, Sunnyvale, CA) at 450 nm optical density and final concentration of the samples was calculated using SoftMax Pro 5.4 (Molecular Devices, Sunnyvale, CA) via standard curves for reference.

### Statistical analyses

Repeated measures ANOVAs were performed to assess group, time, and group by time interactions with a significant p-value considered as ≤0.05. A Fisher LSD *post*-*hoc* analysis was used to locate differences. Significant interactions were further analyzed using dependent and independent T-tests for differences between time and group, respectively. Observed power has been included for variables with a significant interaction. Statistica (Version 10, Statsoft, Tulsa, OK) was used for all statistical analyses.

## Results

### Baseline, diet, and training volume measurements

No significant differences were present at baseline for the means or variances of the measured variables (*p* > 0.05), nor were any differences found for total calories, carbohydrates, proteins, or fats consumed (*p* > 0.05). Total training volume (weight x repetitions x sets) was not different between groups when analyzed as a gross score or by weekly averages (*p* > 0.05).

### Standard resistance training phase

Squat, BP, and deadlift 1RMs as well as total strength significantly increased in both groups from baseline to week 8 (*p* < 0.05). However, no group x time interactions were found for strength variables in this phase (*p* > 0.05). Vertical jump peak power and BP peak power significantly increased in both groups from baseline to week 8, and vertical jump peak velocity also increased in PLA during this time (*p* < 0.05), while TRT did not (*p* > 0.05). However, no group x time interactions were found for power variables in this phase (*p* > 0.05). A trend was observed for irisin (*p* = 0.061). Analysis of the delta values indicate irisin decreased to a greater extent in PLA versus TRT from week 4 to week 8 (*p* < 0.05), but no group x time interaction was detected (*p* = 0.06).

### Overreach and taper phases

**Fig. 1:**
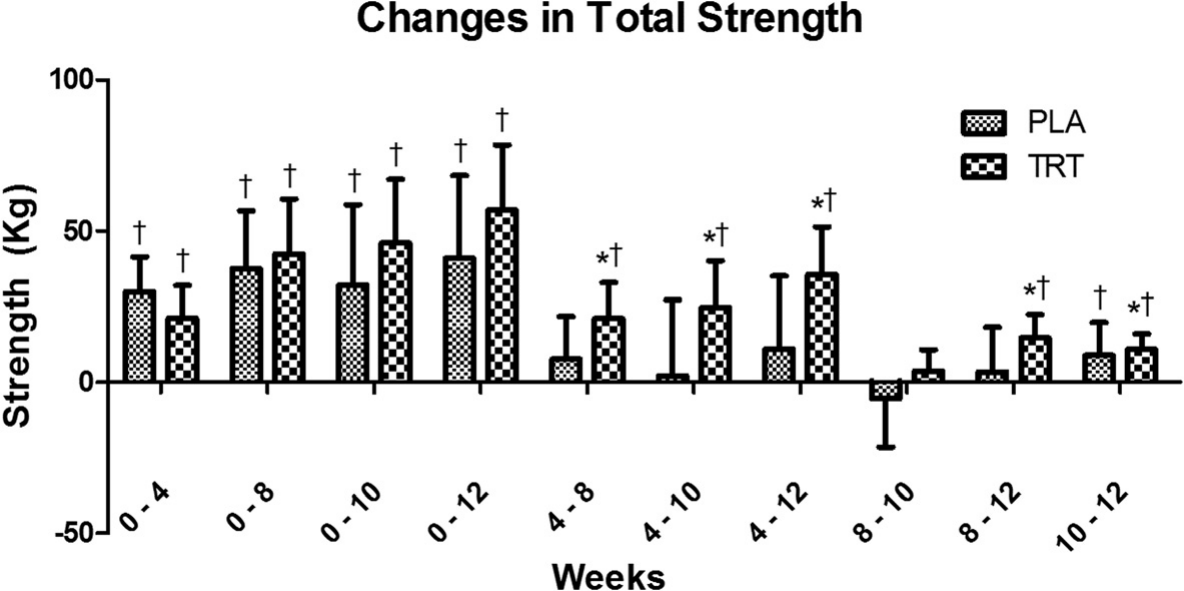
Changes in Total Strength. Delta values between corresponding weeks are presented as mean ± standard deviation. * indicates significantly different from PLA. † indicates a significant within-group change (*p* < 0.05)

**Fig. 2:**
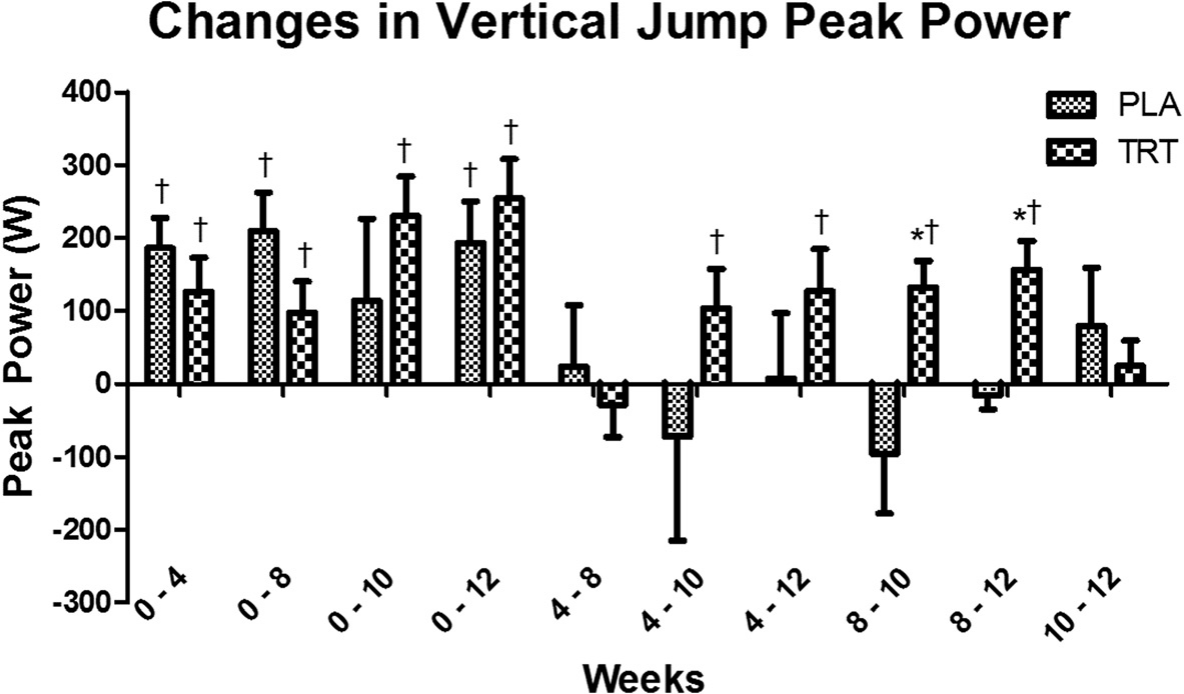
Changes in Vertical Jump Peak Power. Delta values between corresponding weeks are presented as mean ± standard deviation. * indicates significantly different from PLA. † indicates a significant within-group change (*p* < 0.05)

Significant time and group x time interactions (*p* < 0.01) were present for squat and deadlift 1RM as well as total strength (observed power > 0.6809). Squat 1RM increased to a greater extent in TRT than PLA from week 8 to weeks 10 and 12; deadlift increased to a greater extent in TRT than PLA from week 8 to week 12; and total strength increased in TRT versus PLA from week 8 to week 12 (Fig. 1). There were significant time and group x time effects (*p* < 0.05) for vertical jump peak power and peak velocity (observed power > 0.8169). vertical jump peak power significantly (*p* < 0.05) increased in TRT from week 8 to weeks 10 and 12 (Fig. 2), and vertical jump peak velocity increased from weeks 8 to 10, while PLA experienced decrements in both variables at these time points. There were significant time and group x time interactions (*p* < 0.01) for bench press strength endurance and strength endurance total volume (observed power > 0.8229). Wherein, TRT performed more repetitions and total work than PLA at week 10.

### Pre to post

**Fig. 3:**
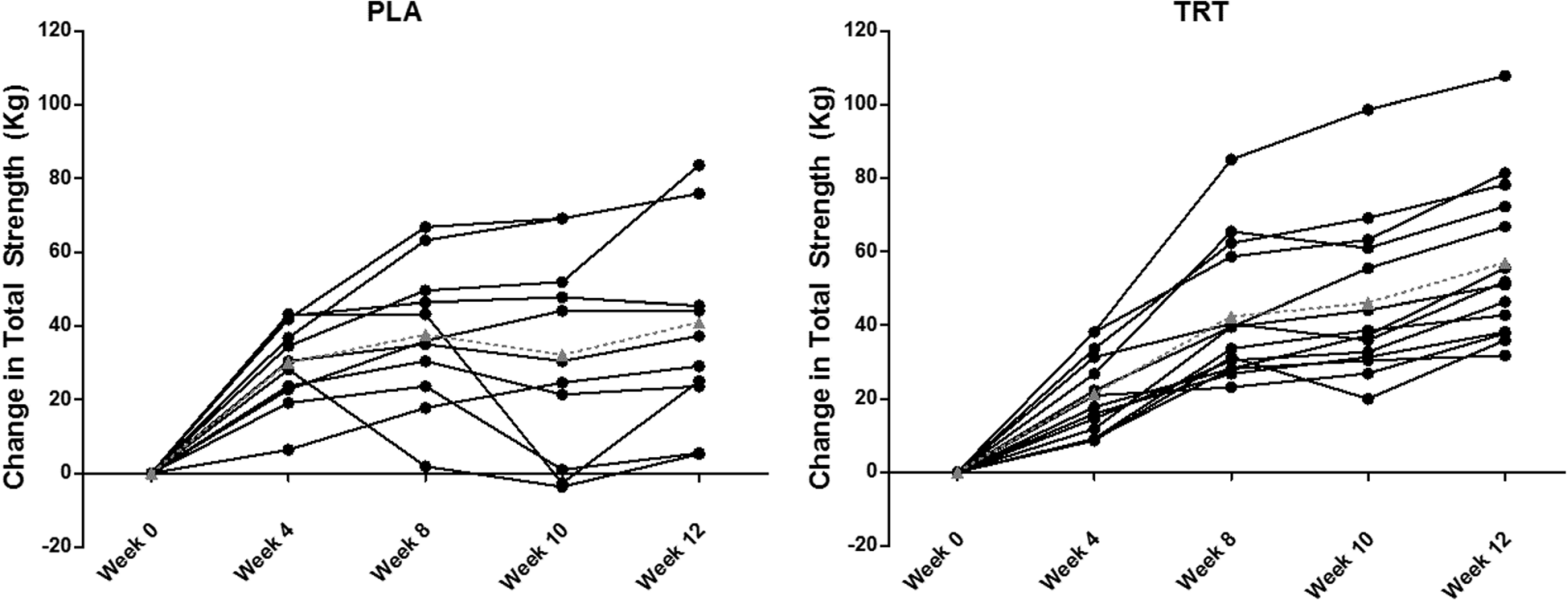
Individual Changes in Total Strength. Data presented represent the mean for each participant at primary time points, corresponding to baseline (Week 0), the mid-point of the standard resistance training phase (Week 4), the end of the standard resistance training phase (Week 8), the end of the overreaching phase (Week 10), and the end of the taper phase (Week 12). The group average is presented as the gray triangle with a dotted line

Significant time and group x time interactions (*p* < 0.05) were found for deadlift 1RM, which increased to a greater extent in TRT than PLA from week 0 to week 12 (observed power = 0.9714). Wingate-determined peak power (*p* = 0.059) and watt:mass (*p* = 0.054) tended to increase to a greater degree in the TRT group. Performance data is presented in Table 1, and blood data is presented in Table 2. Individual changes in total strength are presented in Fig. 3.

**Table 1:** Performance data

Variable	Group	Pre	Week 4	Week 8	Week 10	Post	*p*
Squat 1RM (kg)	PLA	142.8 ± 22.9	153.7 ± 27.1	159.5 ± 25.4	156.1 ± 22.4	158.6 ± 20.9	0.001
	TRT	136.4 ± 25.5	144.6 ± 25.8	152.1 ± 26.9	152. 6 ± 28.5^c^	156.5 ± 27.7^b,c^	
BP 1RM (kg)	PLA	121.5 ± 21.7	127.9 ± 18.1	129.1 ± 18.0	127.5 ± 15.9	131.4 ± 15.0	0.68
	TRT	107.5 ± 17.8	111.4 ± 17.9	116.1 ± 17.5	116.6 ± 17.6	118.9 ± 17.6	
Deadlift lRM (kg)	PLA	174.4 ± 28.3	187.0 ± 26.1	187.6 ± 26.2	187.6 ± 26.2	189.6 ± 24.6	0.008
	TRT	158.1 ± 25.3	167.2 ± 21.8	176.1 ± 18.8^b^	178.8 ± 20.0^b^	183.6 ± 19.1^a,b,c^	
Total Strength (kg)	PLA	438.6 ± 66.3	468.6 ± 65.3	476.2 ± 63.9	470.7 ± 54.9	479.6 ± 52.1	0.009
	TRT	401.9 ± 59.1	423.2 ± 57.0	444.3 ± 54.7^b^	448.0 ± 57.7^b^	458.9 ± 55.6^b,c^	
BP Peak Power (W)	PLA	679.5 ± 123.4	734.2 ± 105.8	748.4 ± 96.3	744.0 ± 85.8	754.5 ± 103.1	0.91
	TRT	636.9 ± 99.4	671.5 ± 106.2	697.5 ± 104.1	708.3 ± 113.8	705.7 ± 109.9	
BP Peak Velocity (m/s)	PLA	1.94 ± 0.17	1.92 ± 0.17	1.95 ± 0.16	1.95 ± 0.18	1.98 ± 0.15	0.13
	TRT	2.01 ± 0.16	2.02 ± 0.11	2.04 ± 0.10	2.02 ± 0.12	2.01 ± 0.14	
Vertical Jump Height (in)	PLA	23.2 ± 3.1	24.3 ± 3.1	24.0 ± 2.9	23.8 ± 3.2	24.5 ± 3.5	0.18
	TRT	24.5 ± 2.8	24.8 ± 2.7	24.9 ± 2.9	25.3 ± 2.7	25.7 ± 2.6	
Vertical Jump Peak Power (W)	PLA	2743.1 ± 445.6	2928.6 ± 503.0	2951.8 ± 431.3	2856.6 ± 363.8	2936.0 ± 412.1	0.04
	TRT	2821.0 ± 552.8	2947.4 ± 561.0	2918.4 ± 483.1	3050.6 ± 494.7^c^	3074.8 ± 567.8^c^	
Vertical Jump Peak Velocity (m/s)	PLA	3.30 ± 0.28	3.45 ± 0.31	3.47 ± 0.28	3.35 ± 0.40	3.45 ± 0.32	0.002
	TRT	3.44 ± 0.28	3.52 ± 0.23	3.46 ± 0.18	3.58 ± 0.22^c^	3.54 ± 0.23	
Wingate Peak Power (W)	PLA	1028.8 ± 198.1	972.5 ± 181.9	979.8 ± 172.5	933.4 ± 187.8	985.3 ± 241.5	0.060
	TRT	1089.6 ± 171.1	1055.5 ± 166.2	1066.8 ± 131.4	1099.8 ± 192.0	1092.3 ± 233.1	
Wingate Average Power (W)	PLA	703.5 ± 77.2	687.9 ± 83.9	705.2 ± 108.8	692.1 ± 103.4	685.9 ± 116.0	0.22
	TRT	730.4 ± 87.1	699.7 ± 97.2	740.6 ± 85.5	747.3 ± 90.0	748.1 ± 114.8	
Wingate Watt:Mass	PLA	8.3 ± 0.7	8.0 ± 0.6	8.2 ± 0.9	8.0 ± 1.2	7.6 ± 0.9	0.054
	TRT	8.8 ± 1.2	8.3 ± 1.2	8.7 ± 1.0	8.9 ± 1.0	9.0 ± 1.5	
Wingate Average Speed (km/h)	^PLA^	58.3 ± 2.4	57.8 ± 2.7	58.2 ± 4.0	57.9 ± 3.9	57.7 ± 3.8	0.32
	TRT	59.1 ± 2.8	57.2 ± 4.8	59.4 ± 2.7	59.2 ± 3.0	59.6 ± 3.4	
Squat Strength Endurance (# reps)	PLA	27.5 ± 6.6	28.6 ± 7.4	27.5 ± 8.4	27.5 ± 8.5	30.2 ± 9.2	0.51
	TRT	28.8 ± 8.2	29.6 ± 9.3	29.5 ± 8.8	29.3 ± 7.0	34.4 ± 9.4	
BP Strength Endurance (# reps)	PLA	29.0 ± 3.0	25.7 ± 2.6	26.4 ± 2.9	27.0 ± 3.1	28.8 ± 3.3	0.001
	TRT	26.9 ± 4.5	28.6 ± 4.3	27.1 ± 3.6	27.6 ± 3.2^d^	26.7 ± 3.2	
Sqaut Strength Endurance Volume (kg)	PLA	1930.8 ± 444.4	2154.9 ± 558.1	2114.0 ± 498.8	2083.8 ± 498.3	2340.2 ± 598.5	0.52
	TRT	1967.9 ± 685.6	2135.5 ± 747.2	2257.6 ± 815.8	2245.1 ± 695.8	2704.1 ± 898.2	
BP Strength Endurance Volume (kg)	PLA	1758.4 ± 338.5	1637.5 ± 233.2	1693.1 ± 240.5	1722.9 ± 301.9	1885.6 ± 238.5	0.001
	TRT	1452.9 ± 356.3	1609.01 ± 391.4	1586.9 ± 375.8	1625.4 ± 350.0^d^	1603.3 ± 360.5	

Data are presented as mean ± standard deviation. A significant difference from PLA is indicated by ^a^if different from pre, ^b^if different from week 4, ^c^if different from week 8, and ^d^if different at the corresponding time point. The *p*-value is derived from an ANOVA and representative of an interaction effect for group by time

**Table 2:** Blood data

Variable	Group	PRE	Week 4	Week 8	POST	*p*
Irisin (μg/mL)	PLA	1.30 ± 0.39	1.65 ± 0.65	0.89 ± 0.36	0.79 ± 0.38	0.06
	TRT	1.20 ± 0.35	1.19 ± 0.39	1.07 ± 0.35^a^	0.97 ± 0.50	
IL-6 (pg/mL)	PLA	1.57 ± 2.62	1.39 ± 0.57	1.20 ± 0.67	1.04 ± 0.56	0.52
	TRT	2.29 ± 3.65	1.55 ± 1.56	1.42 ± 0.83	0.95 ± 0.58	
IL-15 (pg/mL)	PLA	2.21 ± 0.49	2.8 ± 0.64	2.68 ± 0.66	3.36 ± 0.94	0.61
	TRT	2.15 ± 0.48	2.54 ± 0.66	2.41 ± 0.69	3.11 ± 0.83	
FGF-21 (pg/mL)	PLA	80.1 ± 14.9	119.0 ± 34.1	106.5 ± 7.51	81.8 ± 4.6	0.41
	TRT	75.5 ± 7.5	116.8 ± 23.9	104.2 ± 8.5	105.0 ± 76.3	
Myonectin (ng/mL)	PLA	0.26 ± 0.14	0.21 ± 0.08	0.20 ± 0.13	0.36 ± 0.46	0.58
	TRT	0.81 ± 1.52	0.70 ± 1.34	0.64 ± 1.2	0.99 ± 2.05	
Cortisol (ng/mL)	PLA	39.0 ± 20.5	64.0 ± 27.5	70.7 ± 29.5	53.1 ± 19.1	0.11
	TRT	47.0 ± 22.0	43.4 ± 25.8	63.3 ± 27.6	53.3 ± 15.1	
CRP (ng/mL)	PLA	18.1 ± 38.8	11.8 ± 13.0	8.1 ± 9.6	9.3 ± 8.9	0.43
	TRT	11.0 ± 14.2	17.4 ± 25.2	10.0 ± 14.4	6.0 ± 7.5	
GDF-11 (ng/mL)	PLA	0.99 ± 0.58	1.11 ± 0.83	0.49 ± 0.40	0.66 ± 0.57	0.19
	TRT	0.61 ± 0.48	0.55 ± 0.53	0.21 ± 0.29	0.37 ± 0.35	

Data are presented as mean ± standard deviation. The *p*-value is derived from an ANOVA and representative of an interaction effect for group by time. A significant difference from PLA is ^a^if different from week 4

## Discussion

In agreement with the hypotheses, the supplement appeared to beneficially augment strength and power adaptations to resistance training and attenuate power loss during overreaching. Squat and deadlift 1RM both increased to a greater degree in TRT than in PLA, but no effect was observed for bench press 1RM. However, a lack of effect for bench press did not prevent total strength from significantly increasing. Peak power in the vertical jump seemed to increase as a result of training and supplementation, and vertical jump peak power and peak velocity did not decline following overreaching. Moreover, strength endurance was preserved during overreaching in TRT compared to PLA. Blood markers were measured as an exploratory, secondary measure. While significant interactions were not anticipated, supplementation with a proprietary blend of ancient peat and apple extracts may affect irisin levels. However, further research with blood markers as a main outcome is necessary for confirmation.

It is also imperative to discuss the supplement as it pertains specifically to the overreaching phase of the study. The overreaching phase might be defined as a period of exercise training which increases the training stimulus to an amount that exceeds recovery, and the most sensitive and applicable measures of overreaching are temporary decreases in performance [18]. By temporarily, but largely increasing training volume for 2 weeks from week 8 to week 10 in the present study, significant interactions were found for vertical jump peak power and peak velocity between the TRT and PLA groups, in which TRT increased from the beginning to end of the overreach phase while PLA decreased. While it would be expected for both groups to decrease power output during this phase, only PLA had a small decrease compared to a notable increase in TRT, suggesting that the training stimulus meagerly exceeded normal recovery capacity, but the TRT group was able to adequately recover during this time and experience improvements in performance. This may not only have implications for athletes, but also for military and other situations involving an abnormally great physical demand [19].

While it is possible that circulating irisin mediated some effects of supplementation [20], it is unlikely that it was largely responsible for the present observations. ATP has long been believed to enhance vasodilation and blood flow [21–23], and recently direct ATP supplementation has been reported to enhance the blood flow and vasodilatory response to exercise [24]. This may enhance nutrient delivery and waste product removal, thereby enhancing recovery [25, 26]. Moreover, the present supplement has been reported to enhance mitochondrial ATP production [8, 9]. Thus, it is also possible that the proprietary blend of ancient peat and apple extracts increased intracellular ATP and provided more substrate for the phosphagen energy system, such as the mechanism of supplemental creatine which has well documented effects on strength and power [27, 28]. Although in opposition of this potential mechanism, we did not observe any differences in total training volume between groups apart from a difference in BP strength endurance volume following the overreach phase, so this prospective must also be examined more closely with further research, as increased training volume would be expected with a supplement with a similar mechanism to creatine [12]. Furthermore, the strength and power adaptations observed were likely facilitated through increases in muscle mass [16]. Collectively, increased blood flow may enhance the substrate pool and increased intracellular ATP each create a greater energetic, and possibly anabolic environment through inhibition of adenosine monophosphate kinase [29], which may enhance acute and chronic resistance training performance.

A secondary hypothesis for the present observations could be linked to reactive oxygen species and oxidative stress, which ancient peat and apple extracts may reduce [8]. Previous research with flavanol- and/or catechin-based ingredients have reported improved athletic performance markers, potentially due to a reduction in oxidative stress and improved mitochondrial function. Epigallocatechin-3-gallate supplementation has previously demonstrated efficacy for improving maximal oxygen consumption [30], and green tea extracts have reduced peroxide formation in response to exercise [31]. Therefore, ancient peat and apple extracts may have an antioxidant effect, lending insight to the ingredient’s potential to improve fatigue resistance.

The present study is in agreement with previous reports on direct ATP supplementation and the resistance training-induced effects on performance variables. Wherein, strength increased as a result of supplementation. Similarly, vertical jump peak power was preserved during the overreach phase. However, the proprietary blend of ancient peat and apple extracts may be better suited to resist performance decrements, as a decline in performance was only observed in the PLA group in the present study, whereas both the supplemented and placebo groups declined in previous observations of direct ATP supplementation [32]. Additionally, the present study observed an increase in Wingate watt:mass ratio, indicating the TRT group may have increased power output independent of any potential changes in body composition.

While it may not be appropriate to compare direct and indirect ATP enhancement due to their dissimilar compositions and dosage levels, and because their respective mechanisms of action are likely different, the ergogenic effects of direct ATP supplementation have also been reported in different settings. Rathmacher et al. [33] observed an effect of supplementation for improving set 2 minimum peak torque and decreasing set 3 muscle fatigue. Moreover, Jordan et al. [3] indicated beneficial effects for total repetitions and total volume performed following 2 weeks supplementation with oral ATP. Each of these studies supports the present findings of an effect for bench press strength endurance. In the present study, bench press strength endurance only reached significance following the overreach phase, suggesting supplementation maintains strength endurance. No effects were observed for squat strength endurance, and this may be due to a more rapid increase in squat 1RM compared to BP 1RM resulting in an inability to maintain work output. Moreover, the lack of effects observed for upper body strength and power is more likely a result of the lower-body emphasis of training versus a local effect of supplementation. The present study is also limited by the volume of performance testing performed within a single day, such that variables from the strength endurance or Wingate tests may have undergone a more robust change than observed due to accumulated fatigue despite the measurements having a high degree of reliability. Moreover, examination of blood or tissue ATP levels would strengthen the present study, as blood or tissue levels of ATP have not be confirmed to increase with chronic use of ancient peat and apple extracts.

## Conclusion

This is the first study examining the ergogenic potential of endogenous ATP enhancement with supplementation. The proprietary blend of ancient peat and apple extracts were capable of increasing lower body and total strength as well as lower body power output compared at an equal-volume, visually-identical placebo. Moreover, the supplement was able to prevent performance decrements commonly associated with increased training volume and overreaching. However, the supplement does not appear to augment cortisol and other blood markers of recovery aside from possibly altering irisin. Future research should seek to evaluate the acute effects of the supplement as well as the effects of the supplement in an endurance exercise setting, as the current purported mechanism is mitochondrial ATP production.

## Abbreviations

1RM, 1 repetition maximum; ATP, adenosine triphosphate; BP, bench press; CRP, C-reactive protein; FGF-21, fibroblast growth factor 21; GDF-11, growth differentiation factor 11; IL-15, interleukin 15; IL-6, interleukin 6; PLA, placebo; TRT, treatment
